# Supplementary material for: Programmed cell removal by calreticulin in tissue homeostasis and cancer
Source: Nat Commun. 2018 Aug 10;9:3194. doi: 10.1038/s41467-018-05211-7 (PMC6086865; doi:10.1038/s41467-018-05211-7)
Supplement: Supplementary file 1 — Supplementary Information [file 41467_2018_5211_MOESM1_ESM.pdf]

Supplementary Information

Feng et al "Programmed cell removal by calreticulin in tissue homeostasis and cancer"

Supplementary Figures 1-9.

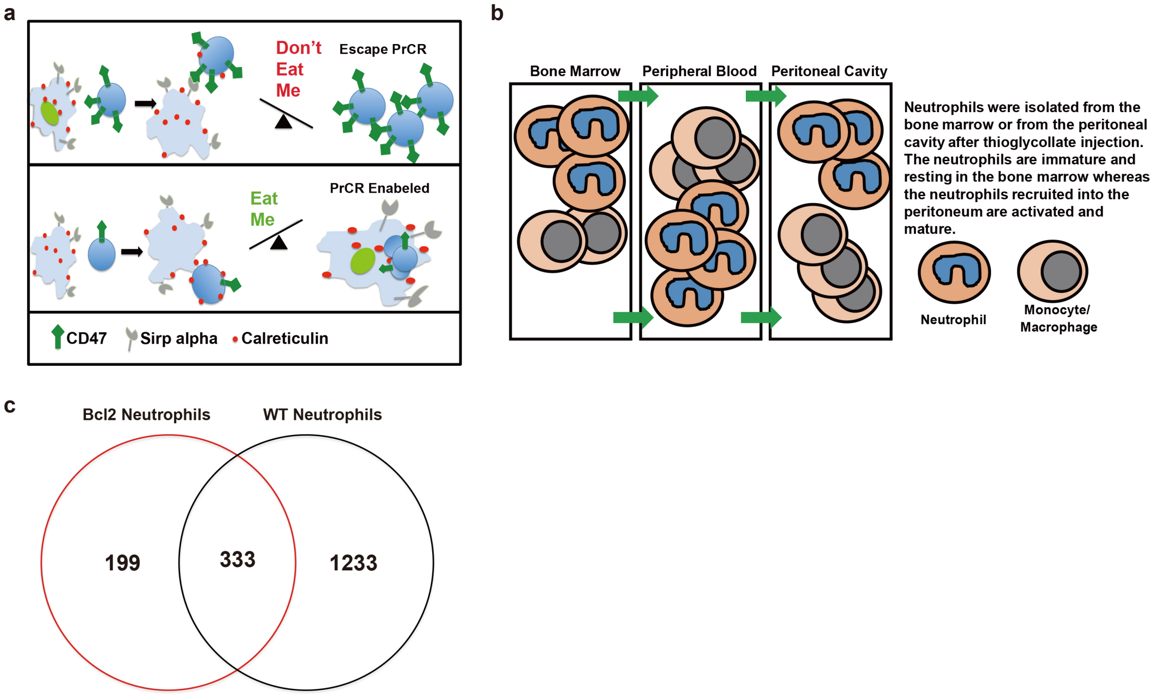

Supplementary Figure 1. A mouse peritonitis model showing programmed cell removal of living cells (a) A schematic figure showing the system of opposing anti- and pro-phagocytic signals affecting macrophage-mediated PrCR. (b) A schematic of migration of neutrophils and monocyte/macrophages after thioglycollate injection. RNAseq data for WT and *MRP8-Bcl2* neutrophils was compared between populations in the bone marrow (immature) and peritoneal cavity (mature). (c) Venn diagram showing the groups of genes that were upregulated in peritoneal vs. bone marrow neutrophils from WT mice (right; susceptible to programmed cell death and programmed cell removal (PrCR)) and *MRP8-Bcl2* mice (left; inhibited programmed cell death but not PrCR). The overalaping 333 genes that increase in expression during neutrophil maturation are potentially PrCR-relevant.

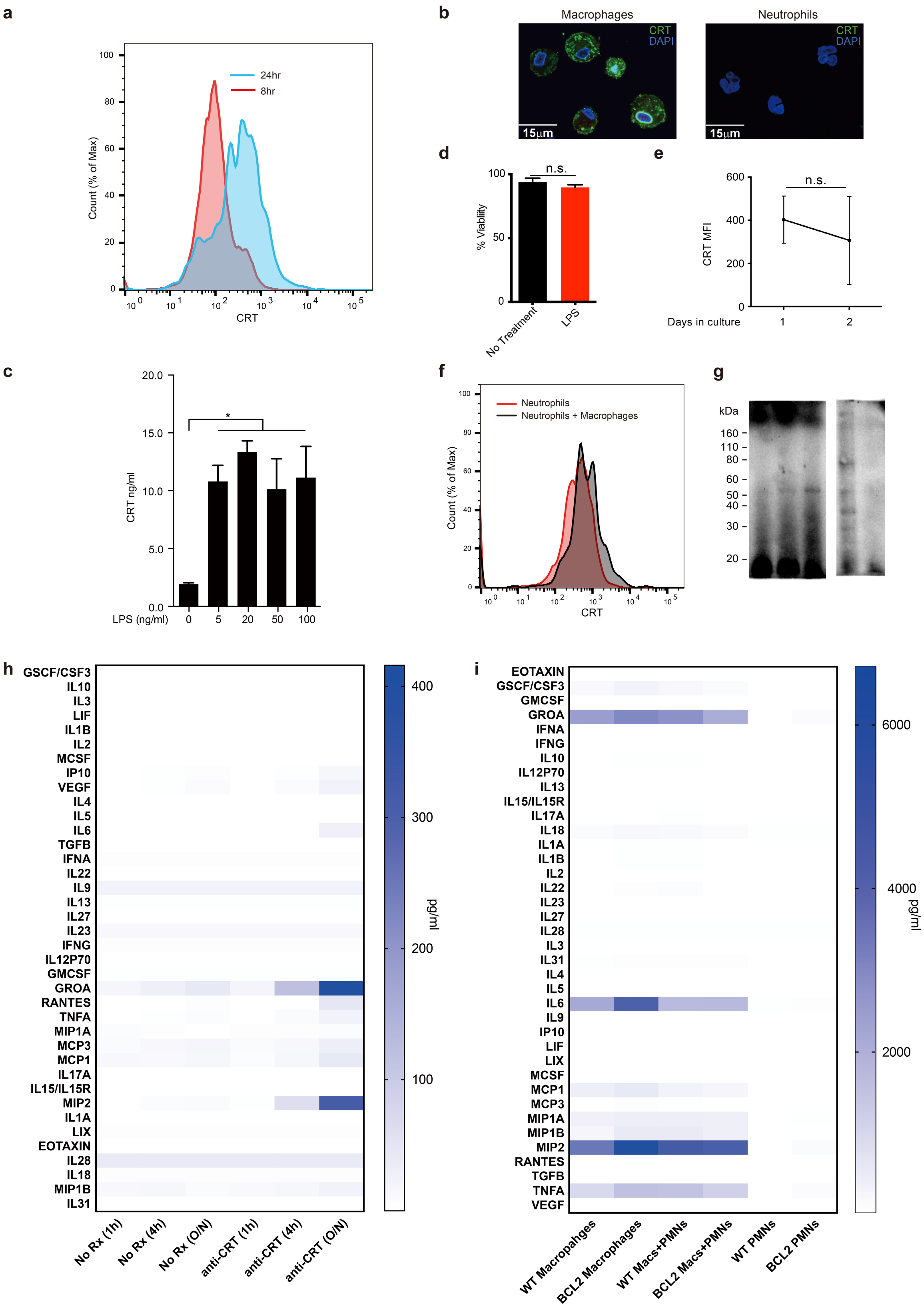

Supplementary Figure 2. Cell surface CRT determines programmed cell removal of neutrophils in peritonitis (a) Cell surface levels of CRT on neutrophils 8 and 24 hrs after

thioglycolate injection, by flow cytometry analysis. (b) Immunofluorescent staining of CRT in human neutrophils and macrophages. CRT is undetectable in neutrophils but abundant in macrophages. CRT localized to perinuclear regions, vesicles and cell surface of macrophages. (c) Secretion of CRT by resting and activated macrophages. ELISA assay showing the amount of CRT in medium with macrophages in culture. Bone marrow-derived macrophages were treated with 0, 5, 20, 50, 100ng ml^{-1} of lipopolysacharide (LPS) overnight for activation. n=3 for each condition. *P<0.05 (*t* test) for CRT secretion between ctrl and LPS treatment. (d) Viability of macrophages in culture. Bone marrow-derived macrophages were treated with 0 or 100ng ml^{-1} of lipopolysacharide (LPS) overnight and collected for viability measurement. Cells that were AnnexinV-DAPI- were considered as viable cells. More than 90% of the cells were viable. n.s. (*t* test), no significant differences in cell viability was observed in control and LPS treated cells. n=3 for each condition. (e) Expression levels of cell surface CRT on neutrophils cultured alone. n.s. (*t* test), no significant differences. CRT levels on neutrophils remained unchanged when cultured alone. (f) Cell surface levels of CRT on neutrophils cultured alone or with macrophages in a 0.4-μm Boyden chamber overnight, assayed by flow cytometry. (g) Full blots of Fig. 3g. (h) Cytokine profiling of supernatant from macrophages treated with CRT blocking antibody for 1h, 4h and overnight (O/N), as examined by luminex assay. (i) Cytokine profiling of supernatant from macrophages and neutrophils alone and in co-culture, as examined by luminex assay. Macrophages and neutrophils were collected from *MRP8-Bcl2* mice as described in "Experimental Procedures". Error bars represent standard deviation in (c), (d) and (e). In (e), MFI, mean fluorescence intensity.

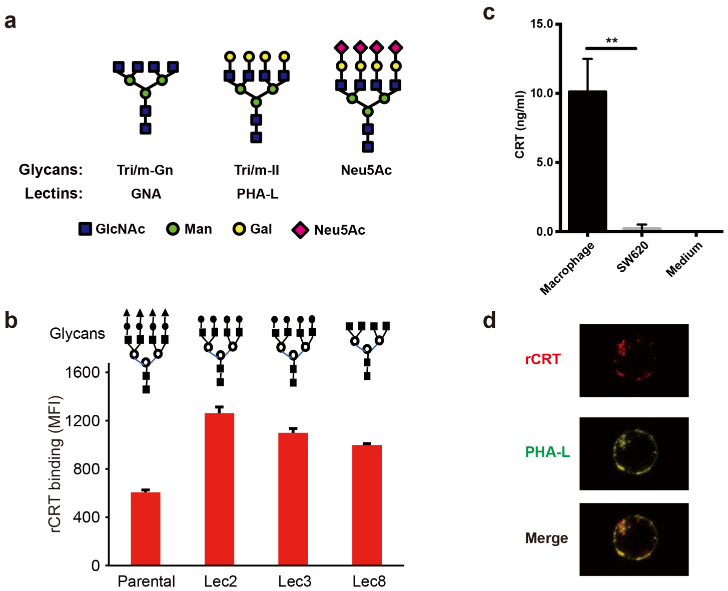

Supplementary Figure 3. CRT binds to cell surface Tri-m/II glycans (a) A schematic of N-glycan "cryptic" glyco-epitopes, Tri/m-II and Tri/m-Gn, that are often expressed by tumor-associated asialo-glycoproteins. GlcNAc, N-Acetylglucosamine; Man, mannose; Gal, Galactose; Neu5Ac, N-Acetylneuraminic acid; GNA, Galanthus nivalis lectin; PHA-L, Phytohaemagglutinin-L. (b) Examination of binding of recombinant CRT (rCRT) to cell surface Tri/m-II and its derivatives. rCRT was incubated with CHO WT and mutant cell lines (Parental, Lec2, Lec3 and Lec8) and its binding was examined by flow cytometry analysis. The major glycan structures (Tri/m-II and its derivatives) by each line were displayed accordingly. (c) ELISA assay showing the amount of CRT in medium (RPMI) with macrophages and SW620 cells. Cells were treated with 100ng ml^{-1} of lipopolysacharide (LPS) overnight. Macrophages but not the cancer cells were able to secrete CRT to the extracellular medium. ** P<0.01 (*t* test) for CRT secretion between macrophages and SW620 cells. (d) Immunofluorescent staining of recombinant CRT (rCRT) and PHA-L in HL60 cells. Immunoflrorescent staining indicated co-localization of rCRT and PHA-L on target cancer cells. Error bars represent standard deviation in (b) and (c).

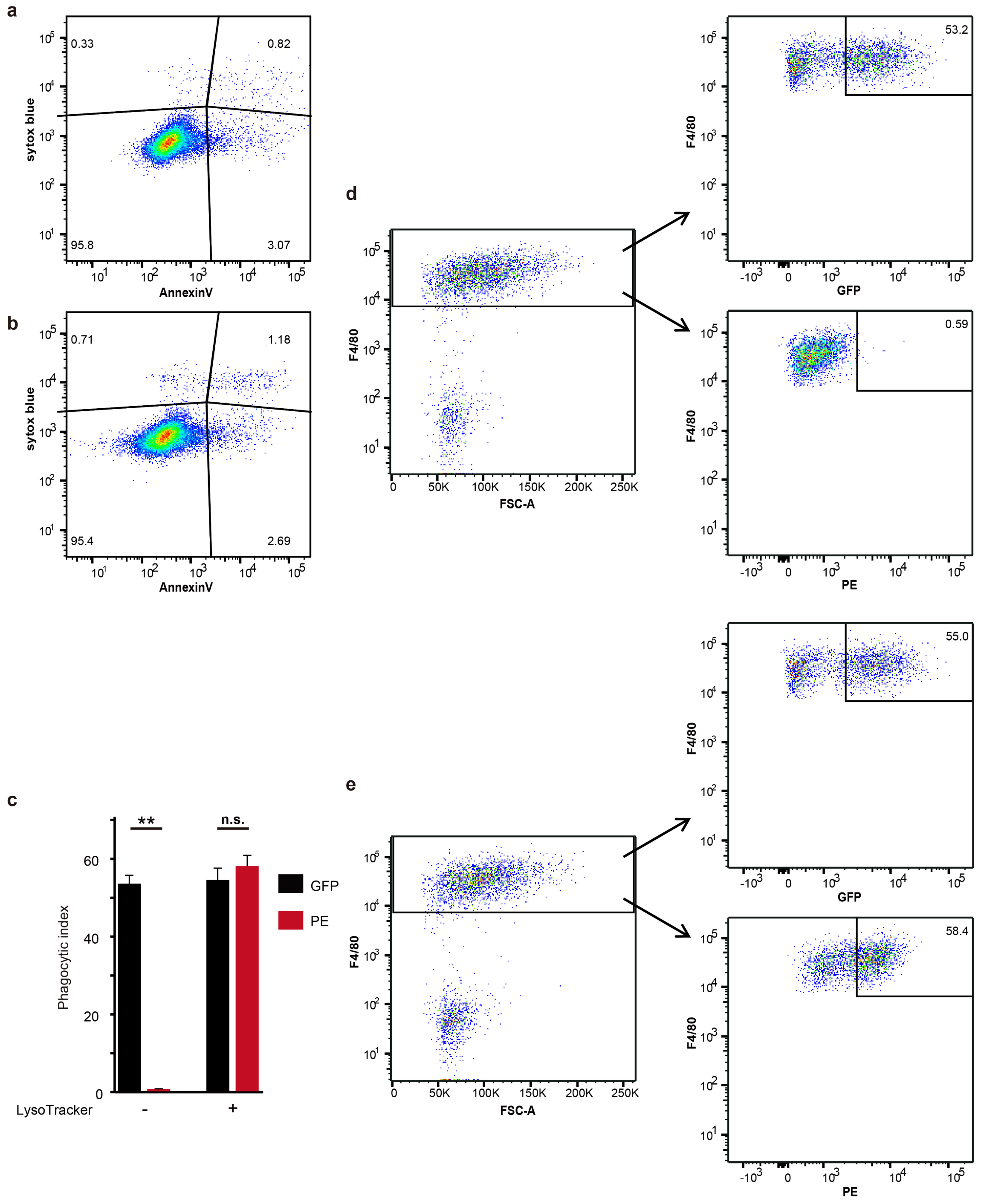

Supplementary Figure 4. Living cancer cells were engulfed by macrophages during PrCR (a) & (b), Measurement of cell viability during PrCR. Target cells used in phagocytosis assay were viable before they were engulfed by macrophages. SW620 cells were collected from the cell culture dish directly (a) or incubated in the experimental condition for phagocytosis assay for 2hrs (b), and cell viability was measured by staining with sytox blue and AnnexinV and analyzed by flow cytometry. Cells that were AnnexinV-sytox blue- were considered as viable cells. More than 95% of the cells were viable and incubation in the experimental condition for phagocytosis assay showed no decrease in cell viability. (c)-(e), Measurement of cellular engulfment of target cancer

cells during PrCR. SW620-GFP cells were treated with or without LysoTracker Red DND-99 and incubated with mouse bone marrow-derived macrophages for phagocytosis. Phagocytic index was defined as the percentage of macrophages that phagocytosed target cells. LysoTracker probes are excited at acidic PH with an emission of 590nm (measured in PE channel). No phagocytosis was detected in PE channel when cells were not pre-loaded with LysoTracker probes. When cells were pre-loaded with LysoTracker probes, phagocytic indices analyzed based on GFP and PE signals showed no difference (c), indicating target cells were engulfed and delivered to acidic organelles for digestion instead of staying attaching to macrophage cell surface. ** $P<0.01$ (*t* test) for phagocytic indices analyzed based on GFP and PE signals without LysoTracker. n.s. (*t* test), no significant differences in phagocytic indices analyzed based on GFP and PE signals when using LysoTracker. (d) and (e) are representative FACS analyses showing macrophages (F4/80+) were analyzed for their phagocytic index based on GFP or PE signals. F4/80+GFP+ and F4/80+PE+ cells are macrophages that phagocytosed SW620-GFP cells or SW620-GFP cells pre-loaded with LysoTracker. Error bars represent standard deviation in (c).

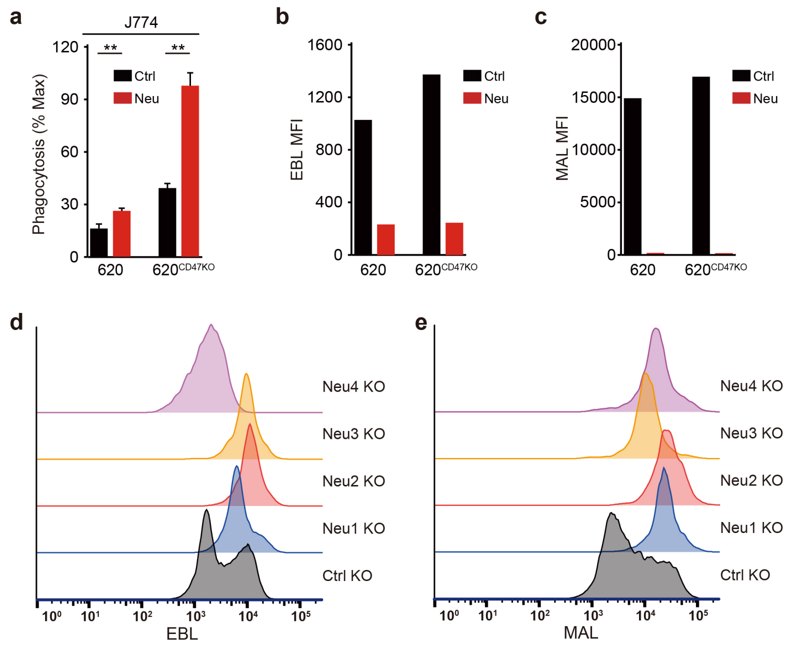

Supplementary Figure 5. Exposure of cell surface asialoglycan epitopes induces PrCR of living cancer cells (a)-(c), Phagocytosis (a) and cell surface sialic acids expression (b and c) of SW620 cells with neuraminidase treatment. Phagocytosis assays were performed with WT and CD47^{KO} SW620 cells as target cells and J774 macrophages. Removal of cell surface sialic acids on SW620 cells by neuraminidase was examined by staining with EBL and MAL by flow cytometry analysis. EBL, Elderberry Bark Lectin; MAL, Maackia Amurensis Lectin II. ** P<0.01 (*t* test) for phagocytosis between control and neuraminidase-treated groups. (d) & (e), Cell surface sialic acids expression on HL60 cells with Neu1-Neu4 gene knockout by CRISPR, as examined by staining with EBL (d) and MAL (e) by flow cytometry analysis. Neu2 and Neu3 knockout enhanced cell surface sialic acids attached with the α2, 6 linkage while knockout of any of the four Neu genes promoted cell surface sialic acids attached with the α2, 3 linkage. Error bars represent standard deviation in (a). In (b) and (c), MFI, mean fluorescence intensity.

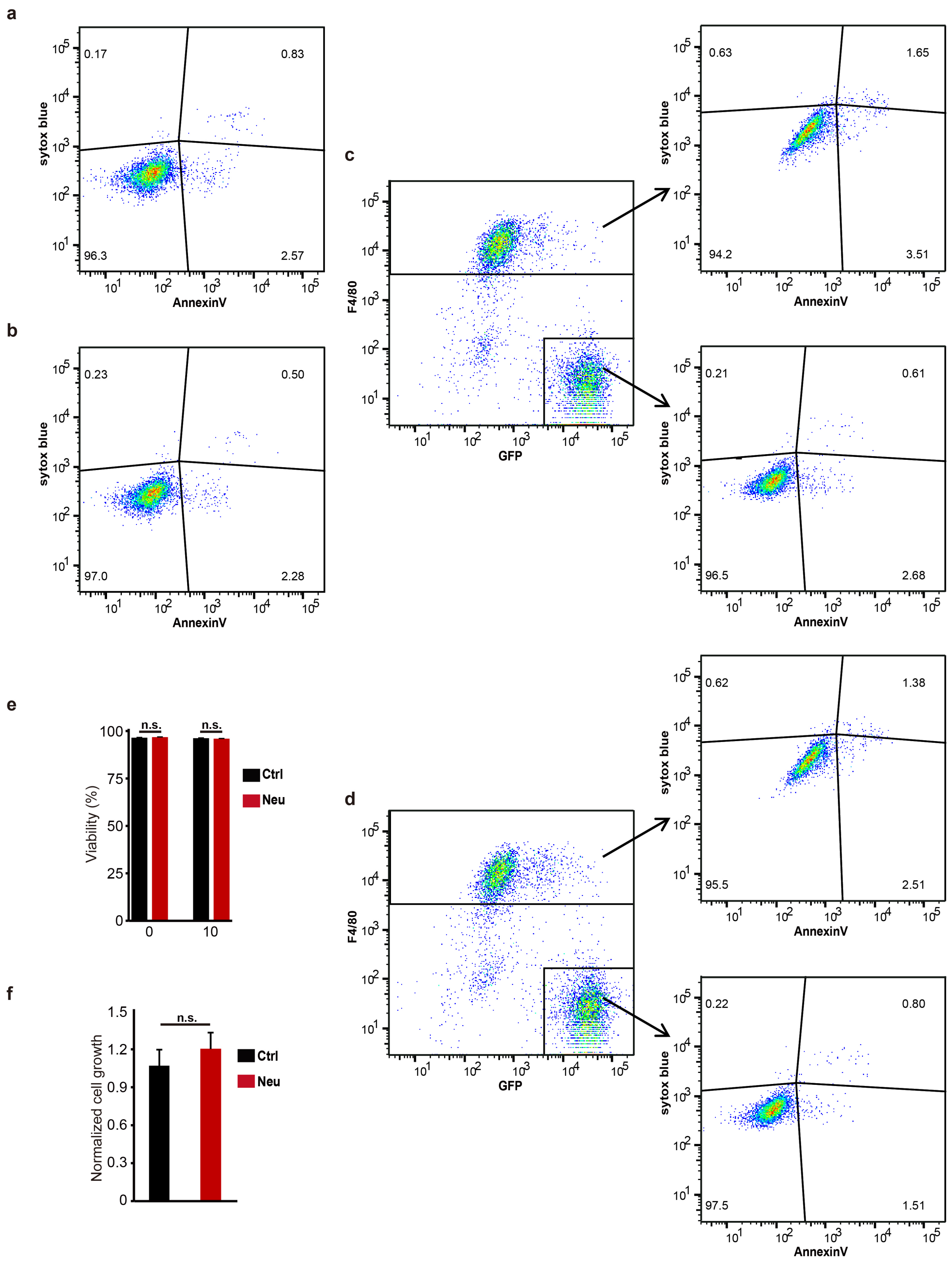

Supplementary Figure 6. Neuraminidase treatment promoted PrCR of living cancer cells but showed no effect on cell proliferation and survival (a)-(d), Measurement of cell viability and proliferation after neuraminidase treatment. SW620-GFP cells were treated with heat inactivated neuraminidase (a) or neuraminidase (b). Cell viability was measured by staining with

sytox blue and AnnexinV and analyzed by flow cytometry. More than 95% of the cells were viable. c & d, representative FACS analyses showing measurement of cell viability after co-culturing macrophages and target cancer cells. Phagocytosis assays were performed with SW620 cells pre-treated with heat inactivated neuraminidase or neuraminidase as target cells. Mouse bone marrow-derived macrophages were used for the assay. After co-culture for 2 hours, macrophages (F4/80+) and surviving cancer cells (GFP+F4/80-) were stained with sytox blue and AnnexinV. Cells that were AnnexinV-sytox blue- were considered as viable cells. More than 95% of macrophages and SW620 cells were viable. Incubation with macrophages did not induce cells death for cancer cells. (e), Cell viability of SW620 cells treated with heat inactivated neuraminidase or neuraminidase. SW620-GFP cells were treated with heat inactivated neuraminidase or neuraminidase. Cell viability was measured immediately after the treatment (Day 0) or after the cells were put back to culture for 10 days. Cells that were AnnexinV-sytox blue- were considered as viable cells. n.s. (*t* test), no significant differences between control and neuramindase-treated groups. More than 95% of the cells were viable, indicating neuraminidase treatment showed no effect in inducing cell death. (f), Cell proliferation of SW620 cells treated with heat inactivated neuraminidase or neuraminidase. SW620-GFP cells were treated with heat inactivated neuraminidase or neuraminidase. Cells after treatment were put back to culture and cell growth was monitored by counting cell numbers. n.s. (*t* test), no significant differences between control and neuramindase-treated groups. After a 10-day culture no significant difference in cell growth was observed between control and neuraminidase-treated cell, indicating neuraminidase treatment showed no effect in inhibiting cell proliferation. Cell growth was normalized to the growth of control group. Error bars represent standard deviation in (e) and (f).

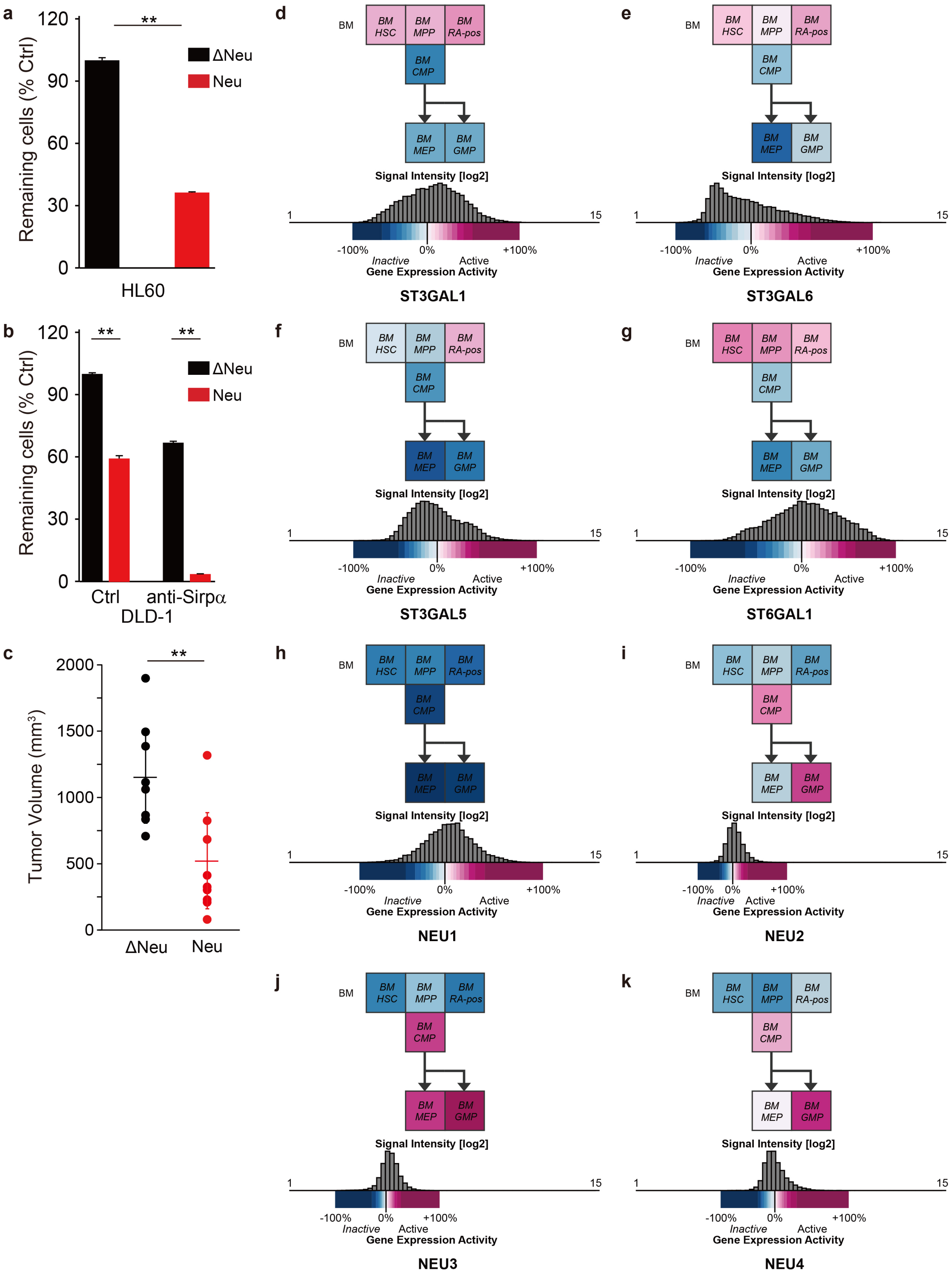

Supplementary Figure 7. Regulation of CRT-binding sites in cancer and hematopoiesis (a) & (b), Phagocytosis of HL60 (a) and DLD-1 (b) cells with neuraminidase treatment. HL60 and DLD-1 cells were treated with heat inactivated neuraminidase or neuraminidase and co-cultured with mouse bone marrow derived macrophages. Remaining cancer cells after overnight co-culture

were examined by flow cytometry. ** P<0.01 (*t* test) for remaining cells between heat inactivated neuraminidase (Δneu)- and neuraminidase (neu)-treated groups. (c), Volume of tumors formed by subcutaneous injection of HL60 cells in NSG mice from the experiment in Figure 6A, as measured on day 28. Treatment with neuraminidases significantly inhibited tumor growth. ** P<0.01 (*t* test) for volume of tumors between heat inactivated neuraminidase (Δneu)- and neuraminidase (neu)-treated groups. (d)-(k), Genes related to the regulation of CRT-binding sites in hematopoietic stem cells (HSCs) and their differentiated progeny (i.e., progenitor cells). Schematics generated with Gene Expression Commons showed upregulation of sialyltransferases pathways and downregulation of neuraminidases pathways in HSCs, which led to downregulation of CRT-binding sites in HSCs, as compared to the downstream cells with higher turnover rates. BM, Bone Marrow; HSC, Hematopoietic Stem Cell; MPP, Multipotential Progenitor; RA-pos, CD45RA+ Multipotential Progenitor; CMP, Common Myeloid Progenitor; MEP, Megakaryocyte-erythroid Progenitor; GMP, Granulocyte-Macrophage Progenitor.

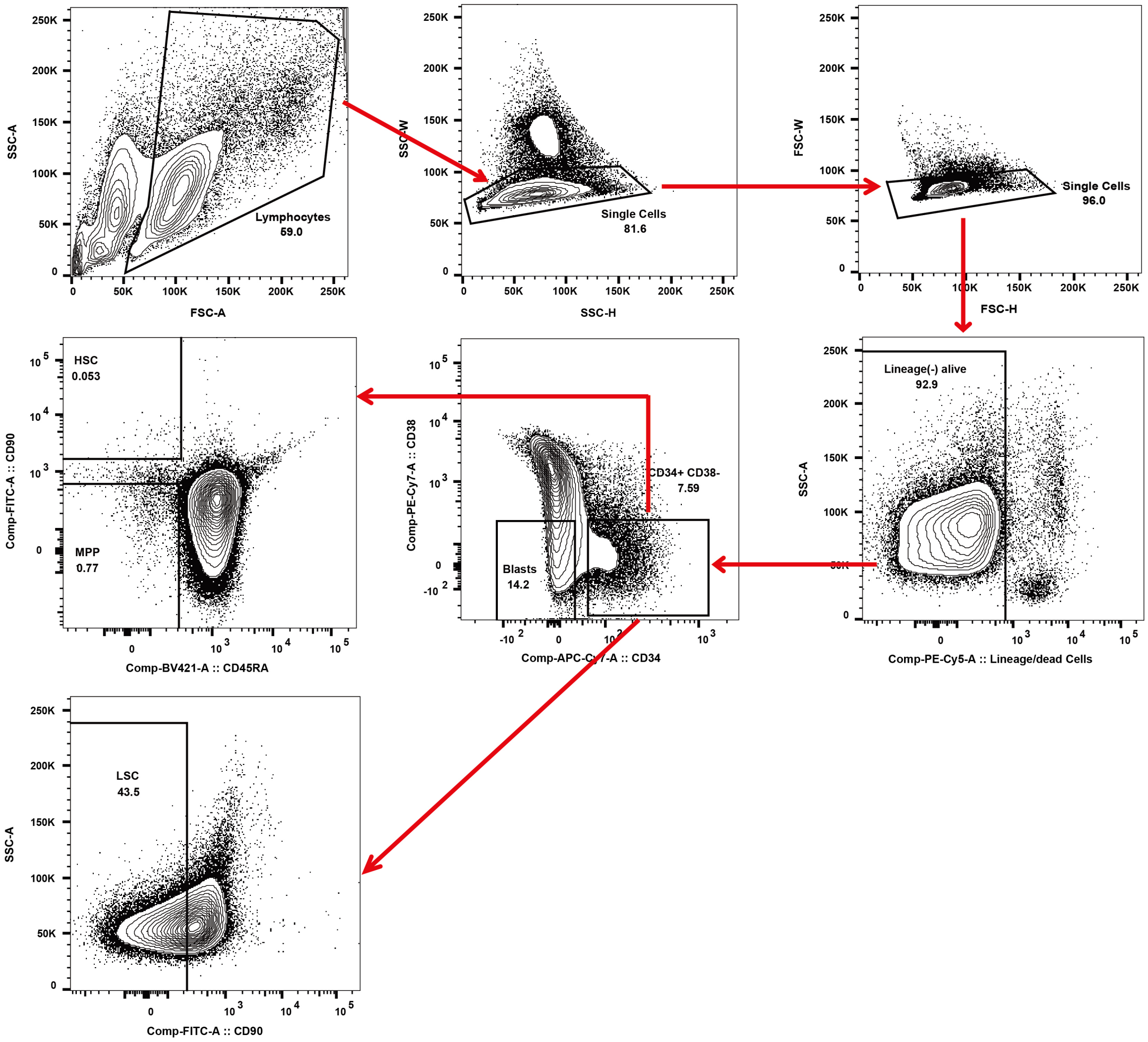

Supplementary Figure 8. Analysis of primary human AML samples Cell populations were identified including HSC ($Lin^{-}CD34^{+}CD38^{-}CD90^{+}CD45RA^{-}$), MPP ($Lin^{-}CD34^{+}CD38^{-}CD90^{-}CD45RA^{-}$), LSC ($Lin^{-}CD34^{+}CD38^{-}CD90^{-}$), and Blasts ($Lin^{-}CD34^{-}$). A representative experiment was shown for identifying different populations.

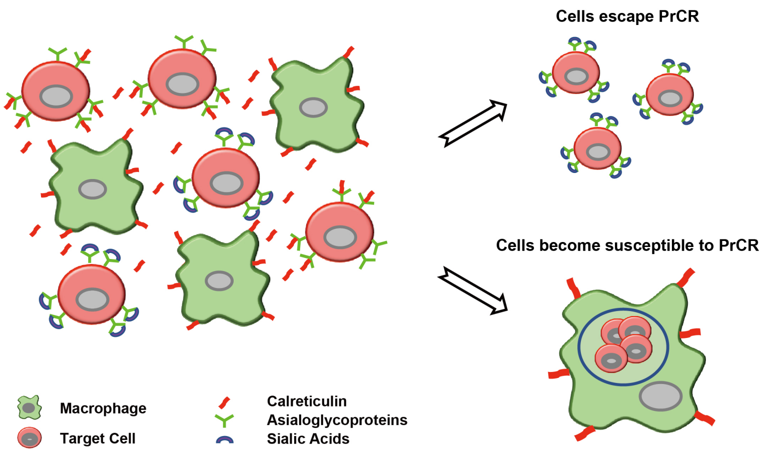

Supplementary Figure 9. An overall diagram demonstrating the regulation of sialic acid dictates CRT binding and phagocytosis During programmed cell removal, unwanted cells with CRT-binding sites available are recognized by macrophages and labeled with CRT to initiate phagocytosis. Cells without CRT-binding ligands expression or with sialic acids upregulation masking such ligands are able to escape from PrCR.
